# The use of telehealth for diabetes management: a qualitative study of telehealth provider perceptions

**DOI:** 10.1186/1748-5908-2-14

**Published:** 2007-05-02

**Authors:** Faith P Hopp, Mary M Hogan, Peter A Woodbridge, Julie C Lowery

**Affiliations:** 1 VA HSR&D Center for Practice Management and Outcomes Research, VA Ann Arbor Health Care System, Ann Arbor, MI, USA; 2 School of Social Work, Wayne State University, Detroit, MI, USA; 3 VA HSR&D Center on Implementing Evidence-Based Practice, Richard L. Roudebush VA Medical Center, Indianapolis, IN, USA; 4 Division of General Internal Medicine and Geriatrics, Department of Medicine, Indiana University School of Medicine, Indianapolis, IN, USA; 5 Department of Pathology and Laboratory Medicine, Indiana University School of Medicine, Indianapolis, IN, USA; 6 University of Michigan School of Public Health, Department of Health Management and Policy, Ann Arbor, MI, USA

## Abstract

**Background:**

Monitoring and Messaging Devices (MMDs) are telehealth systems used by patients in their homes, and are designed to promote patient self-management, patient education, and clinical monitoring and follow-up activities. Although these systems have been widely promoted by health care systems, including the Veterans Health Administration, very little information is available on factors that facilitate use of the MMD system, or on barriers to use.

**Methods:**

We conducted in-depth qualitative interviews with clinicians using MMD-based telehealth programs at two Veterans Affairs Medical Centers in the Midwestern United States.

**Results:**

Findings suggest that MMD program enrollment is limited by both clinical and non-clinical factors, and that patients have varying levels of program participation and system use. Telehealth providers see MMDs as a useful tool for monitoring patients who are interested in working on management of their disease, but are concerned with technical challenges and the time commitment required to use MMDs.

**Conclusion:**

Telehealth includes a rapidly evolving and potentially promising range of technologies for meeting the growing number of patients and clinicians who face the challenges of diabetes care, and future research should explore the most effective means of ensuring successful program implementation.

## Background

Diabetes is a condition with serious consequences for morbidity, mortality, and the use of health care resources. Many persons with diabetes struggle to maintain self-management practices such as attending to glucose levels, blood pressure, and medication management [1-4]. Home-based interventions that make use of innovative communication technologies have been suggested as a means of improving diabetes management [5,6], and home telehealth programs have been promoted as a means of improving chronic care management by offering patient education, promoting self-care practices, and more convenient and frequent monitoring than is typically available through regular office and phone contacts [7-11]. The Veterans Health Administration (VHA) is particularly interested in using telehealth technologies as part of a larger care management strategy designed to facilitate access to care and health improvements with the goal of "providing the right care in the right place at the right time" [8]. As part of this effort, many VA medical centers are using monitoring and messaging devices (MMDs). These are table-top devices designed for home use that require patients to respond to pre-scripted questions and provide educational and self-management content.

Although telehealth interventions have been shown to be both feasible and acceptable, evidence for the effectiveness of these programs in improving condition-specific outcomes remains limited [12]. For example, a recent randomized trial compared the use of MMDs with usual telephone nursing services for heart failure patients and found that MMD use was associated with enhanced self-confidence related to disease management. However, no differences were observed between the two groups in functional status, depression, or quality of life [10]. A recent VA study examined 12-month outcomes for diabetic patients participating in a care management program involving the provision of telehealth services (including televideo and MMD devices), compared with a matched group not receiving these services. Participation in the telehealth program was associated with significantly greater increases in newly scheduled primary care visits that allow the veteran to be seen "just in time", and with insignificant declines in hospital admissions and days of care. A subgroup analysis controlling for available HbA1c data found that the telehealth intervention was associated with fewer hospitalizations relative to the comparison group [13].

A recent qualitative analysis of interviews with staff members at VHA hospitals who used both MMDs and video equipment for patients with a variety of chronic conditions revealed enthusiasm for the continued growth of telehealth-mediated care management programs (including MMD programs) among telehealth providers and referring physicians. However, administrative concerns included the need to balance telehealth funding priorities with other clinical programs amid continued fiscal constraints. Both telehealth providers and referring physicians mentioned the need for information on MMDs and how they are being used for patient care. Telehealth providers stressed the importance of having well developed programs, with resources for successful implementation of programs at multiple hospital sites [14].

The above results suggest a need for deeper understanding of how MMDs are used in practice to identify the factors that facilitate implementation of these programs as well as factors that serve as implementation barriers. As the field of telehealth seeks to move beyond demonstration projects and move toward "normalization", defined as the "routinized embedding of telemedicine in everyday clinical practice", there is a need to engage in studies that seek to describe the processes, facilitators, and barriers to successful implementation [15]. To address these issues, we conducted interviews with telehealth providers, defined as hospital staff members who work directly with patients using MMDs. This study was funded by the VA Diabetes Quality Enhancement Research Initiative (DM-QUERI), which is charged with identifying important problems in diabetes care and the development and evaluation of diabetes care improvement strategies.

The main objective of this study was to obtain information from persons directly providing telehealth services on how the MMD system is used for diabetes care, and to describe barriers and facilitators to implementing MMD programs within the VA health system. Because many patients in the VA medical system have diabetes (primarily type II), we chose to focus on diabetes as our condition of interest. The specific research questions for this study were as follows:

1. How do telehealth providers use the MMD system for diabetes management?

2. What are telehealth providers' perceptions regarding how MMD systems are used by patients with diabetes?

3. What are the views of telehealth providers concerning the role of the MMD system for diabetes care?

## Methods

This study involved semi-structured qualitative interviews with telehealth providers who work directly with diabetes patients using MMDs at two VHA facilities located in Michigan and Indiana. The hospital-based institutional review boards approved the study for the two participating facilities.

### Setting

The MMD program involves having patients use an electronic tabletop device in their homes as one component of their diabetes care management. Patients turn on the machine and respond to text questions, including questions that provide information to the telehealth providers about how they are feeling and their blood sugar results, as well as questions designed for education.

### Sampling

We used a purposive, non-probabilistic sampling methodology [16-18]. as a means of obtaining in-depth information on the use of MMDs for persons with diabetes from the perspective of telehealth providers who were most directly involved in the use of this technology as a component of patient care. Our selection of interview subjects was based on the identification of "typical cases" (clinicians who regularly used MMDs as part of their practice) as a means of obtaining "information rich" data to learn how the MMDs were used with patients. Therefore, we identified those persons who used MMDs with diabetes patients at two sites in our region where MMDs were used regularly as part of patient care. The administrators of the participating facilities provided the research team with lists of telehealth providers who used the MMD systems for diabetes care. These included ten registered nurses, four of whom were also nurse practitioners. The participants included those providing MMD services under two program auspices: outpatient clinic care and home care programs. All telehealth providers who were identified using the purposing sampling methodology agreed to participate in the interviews.

### Measurements

The first two authors conducted in-depth semi-structured interviews with study participants. Interviews lasted from 30 to 90 minutes. One person conducted each interview, and occasionally the second person was present to enhance consistency in interviewing style and questions. Participants were asked about the process of enrolling patients into their programs (outpatient care management or home care), the way in which decisions were made concerning the use of MMDs by the telehealth providers and patients, and the criteria and procedures for discharge from these programs. Respondents were also asked about their opinions on the role of MMDs in care management, on barriers and facilitators to MMD use, and on recommendations for future implementation of MMD programs. The interview format was modified as needed to explore particular areas of interest to the telehealth providers and to accommodate time limitations of some participants.

### Analysis

Interviews were audio taped and transcribed, and two readers (FPH and MMH) developed the codes to organize major content areas of interest. Codes closely mirrored the research question content areas. Once agreement was reached about the codes, each transcript was reviewed and coded by one reader (MH). Two readers (MH and FH) then reviewed two of the ten interviews, and reached substantial agreement on the interview content for each code. A database (ACCESS 2002) was used to organize the coded text from the interviews so that text from multiple interviews could be viewed for each code. Summaries for each code were developed and reviewed. The readers then used a narrative analysis framework to independently identify themes within and across codes based on text from the interviews [16]. Through multiple discussions of the narrative accounts provided in the data, the readers came to a consensus on the major themes.

## Results

The following sections review the processes involved with using the MMD system, including referral, enrollment, and discharge processes. The major themes to emerge from the interviews concerning these processes are then presented.

### Description of MMD processes

Patients typically are referred for nursing services (case management or home care), and telehealth providers then decide which of these patients will use the MMDs. Telehealth providers, especially those in outpatient care management, identify Hemoglobin A1c levels of 9.0 or greater (a blood test which indicates that blood glucose has been out of control) as an important enrolment criterion for outpatient care management programs. Home care providers also consider home care patients with diabetes for MMDs if they feel that doing so will be helpful in managing their condition. Both types of providers consider clinical conditions that would prevent MMD participation, such as vision loss and tremors that would prevent patients from using the touch screen. Non-clinical issues are also considered, including the need for patients to have a land-line phone for MMD data transmission, sufficient skills and interest in using the technology, and sufficient motivation to work on diabetes care.

The MMD machines may be configured individually for each patient who chooses to participate by selecting questions from standard batteries of questions in areas such as general health, glucose testing results, weight, self-management, and education. The telehealth providers then provide an orientation for those who agree to participate in the MMD program, including instructions on how to use the touch screen to answer the MMD-generated questions, how often to take blood sugar readings, and how to report these readings using the MMD system. Because the MMD system is not monitored on nights and weekends, the system is not designed to address urgent issues, and this is mentioned to patients at the time of enrollment in the MMD system.

Persons receiving services in the outpatient clinic receive their orientation in the clinic, and are instructed on how to set up the device in their home, while those receiving home care services receive an in-home orientation and hands-on assistance with setting up the device. Patients activate the MMD system by pressing a button that is clearly visible on the device. They are then presented with text questions, and respond by entering information about their clinical condition, such as their overall health and their current blood sugar level. Information entered by patients using the MMD system is uploaded each night, and telehealth providers typically review MMD information during the workday following MMD entry.

The MMD system uses a series of color-coded alerts to summarize a patient's condition and draw attention to potentially concerning information. Telehealth providers reported paying the most attention to what they refer to as "the numbers", with blood sugars and weight as the more commonly monitored items, followed by general health questions and symptoms such as hypoglycemia or hyperglycemia. If further information is required, the telehealth provider contacts the patient. If the problem indicates a possible need for medication adjustments, the telehealth provider contacts the treating clinician.

### Themes related to facilitators and barriers to MMD implementation

#### Telehealth providers play an important role in considering patients for MMD enrollment

The major theme to emerge from discussions of the patient enrollment process concerns the important role that the telehealth providers play in deciding who to enroll in the MMD program. Several telehealth providers mentioned that when the MMD program was first initiated they were expected to enroll nearly all diabetes patients into the program. The telehealth providers related that this resulted in the enrollment of many patients who were not appropriate for the system, or who did not use the system as expected. As the program evolved, they began to use more discretion in deciding what patients to enroll in the MMD program. As explained by one telehealth provider:

"...when we first started out it was like you'd give them a [MMD] machine unless proven otherwise... but then as, it was like we weren't going to get the grant so the pressure was off and then I think this is nursing judgement, you know?".

The evolution of the program resulted in the current practice, which considers a number of clinical and non-clinical factors in MMD enrollment decisions (see Table 1). With regard to clinical factors, one telehealth provider noted that:

**Table 1:** Issues considered by nurses in MMD enrolment

**Clinical issues**
Level of diabetes control
Need for frequent monitoring
Need to adjust medications
Health conditions preventing use (vision, tremors)

**Non Clinical Issues**
Patient interest and preferences
Readiness to work on diabetes control
Willingness to frequently enter information
Technical: need for land line

"I make that decision by the patient's need. If their diabetes is poorly controlled, then you need to use more tools to get them under control... you don't really need it with all your patients with diabetes. You need it on the ones that need extra help".

Non-clinical considerations were noted by another respondent as follows:

"We have had ... patients ... say, "I don't want to have to deal with that ... but if the patients agree with the monitor, then we give it to them...we review their chart ... because we want to know if there's any issue that will be a concern. Like if we know that there's an active drug user in the home ...A lot of them say no, some of them say yes, or I don't know, or they prefer not to use it for a long time, we say, well just give it a try for a month or so and then if you don't like it, you let us know and we'll go to another plan."

Consequently, although all of the respondents reported actively using the MMD system for at least some patients, they noted that MMD patients are currently a small proportion of their clinical caseloads. For example, one telehealth provider mentioned that out of a total caseload of 80 outpatient diabetes patients managed by two telehealth providers, approximately 20 are using the MMD system.

### The MMD system adds to the telehealth provider's workload

A major theme to emerge concerning the use of MMDs was the time required for the use of the MMD system and associated tasks, and the perception that this was associated with an increase in the telehealth provider's workload. Telehealth providers reported that the use of MMDs requires time to set up the machines, monitor the information put in the MMDs on a daily basis, follow-up of alerts through contacting patients, and make periodic reports on patients who are using MMDs. Sometimes considerable time and effort is required to respond to alerts that may be triggered by clinical markers such as elevated glucose levels. For example, a telehealth provider noted that:

"...when we get alerts where we feel like, yeah this has to be followed up. And mostly everyday we get alerts and we have to call patients...we want to know what's happened and then what we do, if there's a blood sugar that is kind of out of whack, we say, you know, "What happened here?".

Telehealth providers also noted that they sometimes have difficulty reaching patients to get more information related to an alert.

Use of the MMD system also requires a time commitment to set up equipment and respond to technical issues and problems. Nearly all of the telehealth providers expressed concerns with the technical aspects of the MMD program. They focused on the time required to address technical and connectivity problems. For example, one respondent noted:

"It's the technology that bogs the nurse...it takes too much time and while the patient might be willing, the nurse has to plan, do I have that kind of time in my schedule because maybe it's a nice modality but it's a time-consuming modality".

The logistics of replacement of malfunctioning MMDs was seen as inconvenient, particularly when patients need to return a malfunctioning MMD to the medical center. Several telehealth providers expressed the opinion that dealing with technical issues is frustrating and that it is not a good use of their time or expertise. They suggested that having a non-nurse handle setup and technical issues would save them considerable time. One home care telehealth provider noted the advantages of having the same person to handle both clinical and technical issues to provide program consistency for patients.

#### Telehealth provider judgment is required in responding to MMD alerts

The responses from the telehealth providers describing their use of the MMD system suggest that a high level of knowledge and judgment is required when addressing MMD alerts. In particular, telehealth providers use a combination of MMD reports, knowledge of the patient's usual condition, as well as previous responses to MMD questions, in deciding on needed action. One telehealth provider noted that for the majority ("75% of the time") of the most critical ("red") alerts they are able to determine that an alert was not a concern because of information already available about a patient. In other cases, telehealth providers called patients for more information. When patients are contacted, the reason for the alert is sometimes easily understood and does not require further action, such as when a patient eats a piece of cake and subsequently has a temporarily elevated glucose level, or when a patient makes an error in entering information into the MMD system. In other cases, alerts are generated by the MMD system that, upon further inquiry, are recognized as important, and require further patient counselling or consultation with the primary care clinician.

#### Telehealth provider observations of patient use of the MMD system

The third research question concerned the way in which patients use the MMD system. Patients use the MMD system by turning on the machine and then answering questions about their health and symptoms. Patients also take measures, such as glucose and weight, using separate tools, and manually enter these values into the MMD system. Because the telehealth providers evaluate patient input and alerts, they are able to observe how often patients use MMDs and what sections they use. Major themes related to patient use are described below.

#### Patients use the MMD system with varying frequency

The telehealth providers expressed the opinion that some minimum frequency of MMD use by patients is necessary for the system to be useful. However, they also indicated that some patients do not use the MMDs at all following enrollment, while other patients used the system for varying lengths of time. Failure to use the MMDs is a common reason for discharge from the MMD programs, and patients are asked to return MMDs that are not being used.

Patient use of the system was not always synonymous with progress, because a few patients were reported as using the system regularly while failing to move towards improved self-management. For example, one telehealth provider noted that:

"I had a patient who loved the monitor but was not doing anything. He just loved to send in the information. He was not working his plan...he did not go to endocrinology when he was scheduled to. He did not follow up with the dietician...he finally went, but nothing happened to the diet"

#### Patients selectively answer the questions on the MMD system

The telehealth providers reported that patients often selectively answer questions on the diabetes module. The extent to which questions are answered often varies by patient. For example, one telehealth provider noted that:

"The ones that are compliant will answer everything that's in there and so it varies with patients. But with the patients that are non-compliant you can get them, at least, to enter the critical data like weight, blood pressure."

Patients who use the system almost always provided glucose monitoring information, and many patients answered questions concerning their general health and weight reporting. In contrast, several of the telehealth providers felt that patients often get "tired of" the education sections because these sections are long and repetitive, particularly for patients who are expected to use the MMD system on a frequent basis. Varying participation is seen on questions related to high and low blood sugar symptoms. The telehealth providers also reported that many patients do not enter information about other illnesses unless they are feeling sick at the time they answer the MMD questions.

### The role of MMDs for diabetes care

Telehealth providers were also asked about their views concerning the role of the MMD system for diabetes care. While some themes emerged through answering questions about the MMD system, telehealth providers were also specifically asked about their opinions of the MMD system. The discussion of this issue with the telehealth providers revealed several themes that are outlined below.

### The MMD system benefits some patients

Most telehealth providers reported that the system was beneficial for patients, particularly those who require frequent monitoring. For example, one telehealth provider noted that:

"...the way I see it as a benefit is that I'm getting information daily which I'm trusting that is current information, accurate. And that will keep me closer to the patients so I can get actions and whatever they need sooner. That's the benefit to me. That's the major benefit."

The system was also seen as facilitating medication changes, resulting in timely and potentially beneficial responses to patient needs. For example, a telehealth provider mentioned a patient who had used the system and was able to get their insulin completely adjusted as a result of the monitoring reports. In another case, a patient was able to go home from the hospital because their clinician knew that the MMD system was available as a means of following his glucose values and insulin usage very closely. Telehealth providers perceived that many patients enjoyed being part of a special program and using a new and different technology, and were motivated by knowing that someone was watching and monitoring was viewed as motivating for some patients

### Care management is the key; MMDs are a "tool"

Many telehealth providers stated that their main role was to help patients manage diabetes, and commented that the MMD system is one of the tools available for achieving this larger objective. For example, one telehealth provider noted that:

"...I think the [MMD] is a tool for care management, is what I think. And I say that because you still need care management whether you have the [MMD] or not, so this is just a tool that we use to assist us in managing the patient..."

Once patients enter information into the MMD system, telehealth providers need to provide the necessary clinical judgment, follow-up, and other care management tasks based on the patient's responses. Thus, for some patients, the MMD systems are an adjunct to usual care management but the telehealth providers expressed the view that these systems do not fundamentally change the essential and often time-consuming work of caring for patients with diabetes. As noted by one telehealth provider, " [the MMD system] is a great tool... if you have the patients that want to use it and use it the way it's meant to be used. "

## Discussion

The interviews with telehealth providers involved with the MMD program provide a better understanding of issues that should be considered when implementing MMD systems to help achieve guideline-driven goals for diabetes care.

### Establish realistic expectations for diabetes telehealth programs

Our first recommendation is to establish realistic expectations concerning the number and type of patients who are likely to participate in MMD programs. Considerable investment has been made in the telehealth deployment and program development in recent years, particularly by large hospital systems such as the VHA. Over time, as described by these telehealth providers, the emphasis on trying to enroll most patients, which existed at the start of the program, has been adjusted based on experience to a more realistic process for selection. The telehealth providers interviewed in this study indicated that patients who are eager to work on diabetes management are believed to be most likely to benefit from use of these systems. The challenge is how best to identify patients who are most likely to benefit from the system using explicit enrollment criteria. Currently the telehealth providers are using their judgment in making decisions on appropriateness, and these judgments need to be made more explicit and measurable to ensure consistency in program enrollment.

The present study suggests that many patients do not use the MMD device assigned to them, or use the system for a short period of time before dropping out of the program. Moreover, those who do participate are often selective in answering questions on the diabetes module. Better information on the extent to which these issues occur is needed for telehealth-related program and policy decisions, and can best be obtained through a close examination of MMD data from large samples of current users. These findings also suggest that research is needed to provide evidence on what MMD generated questions are most likely to help patients accomplish the goals of diabetes management.

### Improve usability of the technology

Implementation of MMD systems will also require ongoing improvements in the usability of the technology. Concerns about technical issues and problems suggests that the usability of the system needs to be improved, in terms of reliability of the technology as well as refinement of the module questions to focus on those that are most useful for patient assessment and monitoring. To inform this effort, a more systematic means of identification and follow-up on technical issues is needed, based on active collaboration between MMD users and vendors, to make modifications to the MMD system that best meet the needs of clinicians and patients. More information is needed on the use of MMD systems to inform refinement of the MMD modules, including documentation of the length of time that patients actively use the MMD system, the number of MMD sessions completed by patients over specified time periods, as well as the type and frequency of questions that are answered by patients using the MMD system.

The MMD system examined in the present study allows patient communication through responses to specific questions, and communication occurs in only one direction (patient to telehealth provider). Moreover, patients were not able to use the system to add additional information that might clarify their answers to questions. This information, if available, could potentially reduce the need for further follow-up phone calls.

### Consider the impact of implementation on nursing workload issues

The discussion of workload issues with the telehealth providers suggests that successful implementation requires careful consideration of staffing configurations. Given the existing workloads of telehealth providers, it appears that in many cases these programs simply cannot be added as new responsibilities to existing staff, and that additional staffing resources, including non-clinical staff dedicated to addressing technical issues, should be considered as a means of improving successful implementation.

### Study limitations

A limitation of the present research is that it is based on interviews with a small sample of MMD users at only two VA facilities in the midwestern United States, and do not necessarily reflect the experience of other VA facilities or the experiences of programs outside the VA. Even within the VA system, programs vary considerably in terms of the staff expertise and the extent to which telehealth programs have been developed and supported by regional VA leadership. A second limitation is that although we used a team of two researchers to independently code the interview transcripts, resources were not available to organize an independent panel of reviewers to independently code and interpret the data. Such additional steps would have provided a further check on the reliability of the coding process [18]. Finally, because it was outside the purpose of this study, we did not interview persons not currently using the MMD system, and such persons might have additional insights into barriers to starting an MMD program. Future research should more fully explore variations in telehealth program practices in different VA regions and across varying service configurations.

## Conclusion

To our knowledge, this study is the first to use qualitative interviews with clinical staff who regularly use MMD systems to care for diabetes patients to delineate the barriers and facilitators to effective use of MMDs for patient centered care. The research was based on a small number of telehealth providers providing telehealth services in two VA medical centers, and consequently, should be viewed as a starting point for further inquiry. In particular, more work is needed to help determine for what types of diabetes patients MMDs are most effective, and the particular program characteristics and technologies that are most effective in meeting the needs of these diabetes patients. Telehealth includes a rapidly evolving and potentially promising range of technologies for meeting the growing number of patients and clinicians who face the challenges of diabetes care, and future research should explore the most effective means of ensuring successful program implementation.

## Competing interests

The author(s) declare that they have no competing interests.

## Authors' contributions

FPH collaborated in developing the interview instrument, conducting qualitative interviews, and analysis of qualitative data. She wrote the first draft of the manuscript, and revisions based on comments from other authors.

MMH collaborated in developing the interview instrument, conducting qualitative interviews, and took a leadership role in the analysis of qualitative data. She made substantial contributions to revisions of the manuscript.

PAW provided consultation and expertise in the development of the interview instrument, and made editorial contributions to the manuscript.

JCL provided consultation in the development of the interview instrument, and made editorial contributions to the manuscript.
